# The “Hit-and-Run” legacy: epigenetic locking and the survival switch in H. pylori-associated gastric cancer

**DOI:** 10.1038/s41420-026-03210-y

**Published:** 2026-06-23

**Authors:** Junjie Sun, Pengtao Hu, Benno Traub, Marko Kornmann, Uwe Knippschild, Jian Lu, Chengyu Lv, Georgios Giamas

**Affiliations:** 1 Department of General Surgery, Nanjing First Hospital, Nanjing Medical University, Nanjing, China; 2 Department of General, Visceral and Transplantation Surgery, Tübingen University Hospital, Tübingen, Germany; 3 Department of General and Visceral Surgery, Ulm University Hospital, Ulm, Germany; 4 International Oncology Institute, The First Affiliated Hospital of Zhejiang Chinese Medical University, Oncology Department of the First Affiliated Hospital of Zhejiang Chinese Medical University, Hangzhou, China

**Keywords:** Gastric cancer, Cancer microenvironment

## Abstract

The mortality associated with gastric cancer (GC) is mostly attributed to metastasis that is resistant to therapy, presenting a therapeutic challenge that frequently continues even after effective eradication of *Helicobacter pylori* (*H. pylori*). This paper addresses the “eradication paradox” by introducing a novel conceptual framework, the “Survival Switch” hypothesis: *H. pylori* infection not only induces a temporary epithelial–mesenchymal transition (EMT) but also stabilizes gastric epithelial cells in a persistent, drug-resistant “Hybrid Epithelial/Mesenchymal (Hybrid E/M) state.” We systematically outline this evolutionary trajectory through three distinct phases: (1) The Igniter: The virulence factor Cytotoxin-associated gene A (CagA) compromises junctional integrity and commandeers YAP/ZEB1 signaling to initiate plasticity; (2) The Lock: A “Hit-and-Run” mechanism establishes “epigenetic scarring,” specifically methyltransferase-like 3 (METTL3)-mediated N^6^-methyladenosine (m^6^A) modifications, that perpetuates the mesenchymal phenotype independently of the bacterial stimulus; and (3) The Niche: An inflammatory ecosystem comprising CAFs and TAMs functions as an extrinsic amplifier, creating a “fail-safe” network that reinforces the hybrid state through paracrine feedback loops. We propose a theoretical paradigm shift in clinical practice from broad-spectrum cytotoxicity to a “Wake-up and Kill” sequential strategy, employing EMT-reversing drugs to activate the survival switch and re-sensitize tumors before chemotherapy. This review presents a precise strategy for overcoming the epigenetic and stromal barriers of refractory GC by incorporating upcoming technologies like liquid biopsy for dynamic stratification and nanocarrier-based RNA delivery.

## FACTS

1. H. pylori infection not only induces a temporary epithelial-mesenchymal transition (EMT) but also stabilizes gastric epithelial cells in a persistent, drug-resistant “hybrid epithelial/mesenchymal (E/M) state”.
2. The virulence factor CagA initiates this plasticity by compromising junctional integrity and commandeering YAP/ZEB1 signaling.
3. METTL3-mediated m6A modifications act as an epigenetic “lock” that perpetuates the mesenchymal phenotype via a “Hit-and-Run” mechanism, independent of the bacterial stimulus.
4. An inflammatory ecosystem comprising CAFs and TAMs functions as an extrinsic amplifier, reinforcing the hybrid state through paracrine feedback loops.

## Open questions

1. What are the precise molecular thresholds that transition transient H. pylori-induced plasticity into a permanent, therapy-resistant epigenetic scar?
2. How can EMT-reversing drugs be optimally sequenced with standard chemotherapy to maximize the efficacy of the “wake-up and kill” strategy in clinical practice?
3. Can emerging technologies, such as liquid biopsy and nanocarrier-based RNA delivery, effectively target the epigenetic and stromal barriers of refractory gastric cancer?

## Introduction: the unresolved paradox of *H. pylori* eradication

### Epidemiology and etiological factors

Cancer constitutes a major and increasing global health challenge, with gastric cancer (GC) positioned fifth in terms of both mortality and incidence globally [1–4]. In 2024, there were more than 1,000,000 newly diagnosed instances of GC, with over 60% of these cases leading to mortality. GC has emerged as the predominant cause of cancer-related mortality in Asian nations [1, 5]. GC is classified into cardia and non-cardia forms anatomically [1, 5]. The preponderance of global incidence is attributed to non-cardia GC, which is most strongly associated with infectious etiology. Epidemiological studies, notably those conducted in China, have established *Helicobacter pylori* (*H. pylori*) infection as the primary driver for the more prevalent non-cardia subtype, contributing to ~60% of all GC cases, despite the fact that cardia GC represents a smaller proportion of cases [6–10]. Although *H. pylori* is a unique carcinogen, only a small percentage of infected hosts will ultimately develop malignancy, indicating the presence of additional mechanisms.

While mortality rates for GC have declined since 1980, existing preventive measures are inadequate and necessitate further enhancement [5]. Eradication therapy of *H. pylori* infection significantly reduces the risk of GC development [5, 11]. A recent trial has demonstrated that the *H. pylori* vaccine can be effectively applied to children of school age [12]. A lower salt diet may decrease the incidence rate of GC and reduce the synergies with *H. pylori* [13]. Some drugs like non-steroidal anti-inflammatory drugs (NSAIDs) and statins can also mitigate risk for GC [14]. High intake of vegetables and fruits is associated with a reduced risk of GC [5]. China has already implemented screening programs that urge people over 40 to undergo regular gastroscopy examinations [15]. Certain perspectives suggest a higher probability of intestinal metaplasia among *H. pylori* carriers, as well as an increased likelihood of this condition advancing to GC [16].

### The histological landscape: tracing the path to plasticity

The Lauren and WHO classifications are the two predominant histological classifications. With Lauren classification, GC may be categorized as intestinal or diffuse; with WHO classification, it may also be classified as papillary, tubular, mucinous, or poorly cohesive [17]. However, the Cancer Genome Atlas (TCGA) categorization identifies four molecular subgroups of GC: chromosomal instability (CIN), GS, microsatellite instability (MSI), and Epstein-Barr virus (EBV)-positive [17, 18]. GC typically goes through several stages: chronic atrophic gastritis (CAG), gastric ulcer, intestinal metaplasia (IM) and atypical hyperplasia [19]. Epigenetic mechanisms in GC include gene mutations, SCNAs, MSI-H, and CpG island methylation [17, 20–22].

**Fig. 1:**
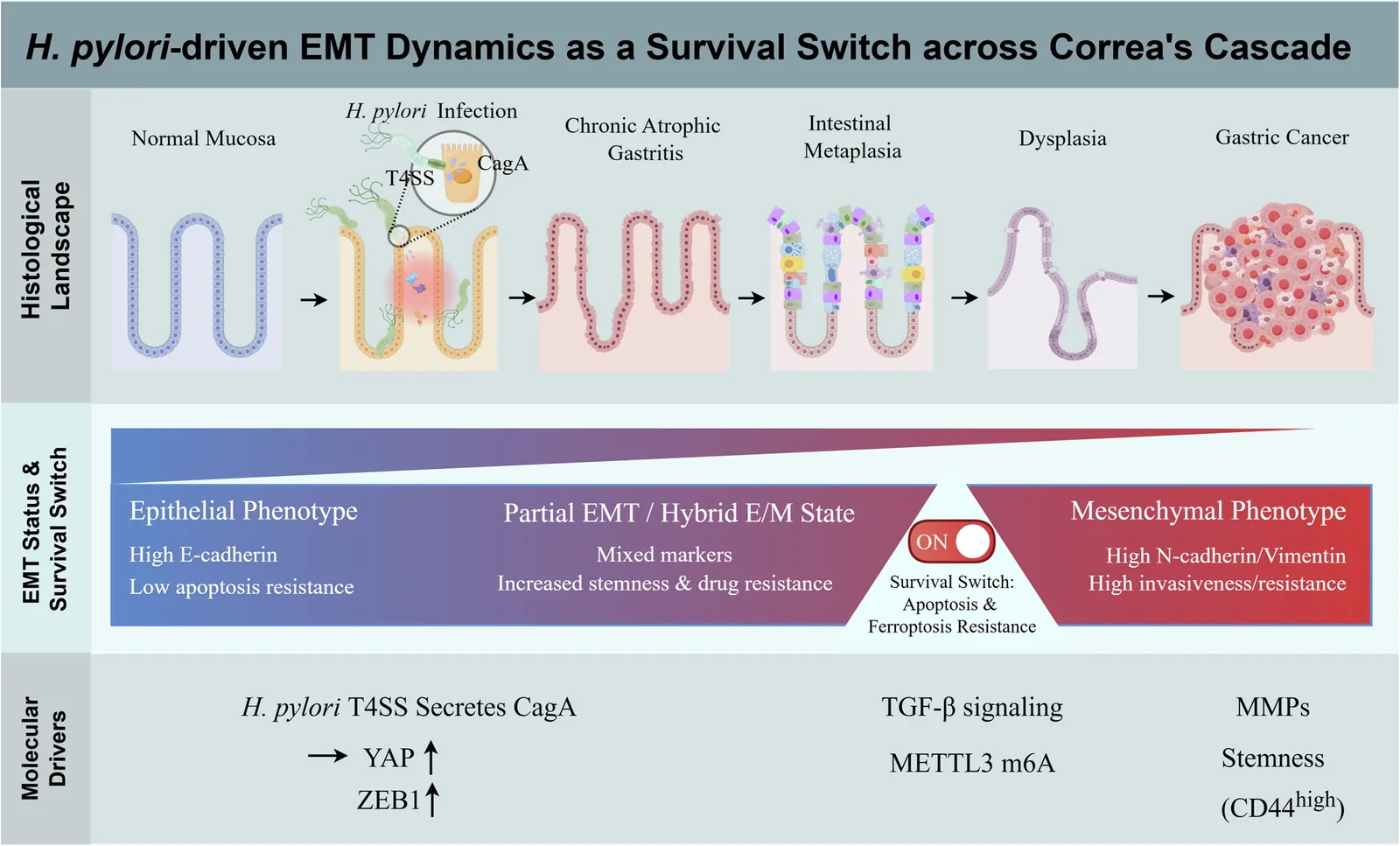
*H. pylori*-driven EMT Dynamics as a Survival Switch across Correa’s Cascade. This diagram incorporates the histological advancement of gastric carcinogenesis with the developing Epithelial-Mesenchymal Plasticity (EMP) and the fundamental molecular determinants. (Top Panel) The histological progression adheres to Correa’s cascade, illustrating the ordered decline from normal mucosa to *H. pylori* infection, chronic atrophic gastritis, intestinal metaplasia, dysplasia, and ultimately GC [23, 24]. Importantly, the hypothesized ‘Survival Switch’ serves as a mechanism that diverges from the traditional epithelial pathway, facilitating the formation of the weakly cohesive GS subtype. (Middle Panel) This trend phenotypically signifies a “Survival Switch”. Instead of a binary transition, *H. pylori* infection induces epithelial cells to enter a stable “Partial EMT” or “Hybrid E/M” state [43, 46]. This hybrid phenotype is critical for disease progression, as it confers cancer stem cell (CSC) properties (e.g., CD44^high^), evasion of apoptosis, and intrinsic drug resistance [44, 48]. (Bottom Panel) The mechanism is initiated by the *H. pylori* virulence factor CagA (translocated by T4SS), which enhances YAP and ZEB1 to disrupt epithelial polarity [52, 64, 101]. In advanced phases (metaplasia to cancer), the mesenchymal state is stabilized by epigenetic remodeling, particularly via TGF-β signaling and Methyltransferase-like 3 (METTL3)-mediated N6-methyladenosine (m^6^A) alteration [81, 83], sustaining the aggressive phenotype independently of the initial bacterial trigger.

The “Correa’s cascade” (Fig. 1) has been considered as the progressive course from CAG to GC [23]. *H. pylori* readily adapts to the acidic environment by producing urease and other mechanisms, then adheres to the surface of the epithelium [24]. The Cytotoxin-associated gene Pathogenicity Island (cag PAI), as a virulence factor, can infiltrate the host cell cytosol, phosphorylate tyrosine, and activate many mitotic signaling pathways, including phosphoinositide 3-kinase/protein kinase B (PI3K/AKT) and mitogen-activated protein kinase kinase/extracellular signal-regulated kinase (MEK/ERK), thus facilitating inflammation and cellular proliferation [24, 25]. Additionally, interactions between *H. pylori* and stem cells result in malignant transformation [24]. In addition, other microbes, aside from *H. pylori*, also contribute to the progression of GC [26, 27]. *Peptostreptococcus* and *Vogesella* have been reported to be enriched in GC [26]. Conversely, lower levels of *H. pylori* are strongly linked to genotoxic microbiologic communities in GC patients [28].

Epstein-Barr virus (EBV)-associated GC is a unique subtype of GC [29]. Zheng et al. developed EBV Net to forecast disease progression stages in EBV-associated GC patients [30]. Tumor heterogeneity results in a composition of various tumor cells with distinct genetic markers, which subsequently influences therapy sensitivity and subtype distribution [31]. In GC, Tumor Protein p53 (TP53) is frequently mutated in CIN. RHOA mutation was mainly identified in diffuse-type GC [17, 32]. Yan et al. found remarkable intratumoral heterogeneity in ERBB2 and FGFR2 overexpression in the GC organoid they developed [32].

Atrophic gastritis (AG) and gastric intestinal metaplasia (GIM), characterized by the reprogramming of gastric epithelial cells to an intestinal phenotype, are predominantly regarded as precancerous lesions of GC (GC) [33, 34]. The activation of 2 homologous caudal-type homeobox genes CDX-1 and CDX-2 is a key step to GIM, and *H. pylori* infection is the most significant cause [33]. Prevention and targeted surveillance of IM are essential [33, 35]. Additionally, intestinal metaplasia has been demonstrated to be the optimal marker of atrophy in stomach mucosa [34]. The reduction of parietal cells caused by atrophy results in the absence of acid and may cause the production of metaplastic mucosa [36].

The molecular landscape delineated by the TCGA has provided more profound insights into the biological determinants of these phenotypes, despite the fact that the Lauren classification remains a cornerstone of clinical pathology [37]. The EMT paradigm is particularly relevant to the GS subtype, which is one of the four molecular subtypes: EBV-positive, MSI, CIN, and GS [37, 38]. The GS subtype, which exhibits a significant overlap with the histologically diffuse-type GC, is particularly enriched for EMT-related gene signatures and pathways that involve cell adhesion and motility (e.g., RHOA mutations and CLDN18-ARHGAP fusions) [39–41]. This “EMT-active” GS subtype is clinically defined as an aggressive phenotype that exhibits intrinsic resistance to standard chemotherapy and a propensity for peritoneal dissemination [42–44]. Consequently, it is not simply academic, but also essential to investigate the molecular cues that induce this EMT state, such as *H. pylori* infection, in order to create precise therapies for this patient subgroup that is notoriously difficult to treat [44].

Although Correa’s cascade establishes the canonical framework for intestinal-type carcinogenesis, it is unable to completely account for the rapid progression or de novo appearance of diffuse-type (GS) tumors. While Correa’s cascade is supported by robust clinical and histological evidence, we hypothesize that our model proposes that the “Survival Switch” operates in a manner that is orthogonal to this cascade. This mechanism is responsible for driving cells away from the organized intestinal phenotype described by Correa and toward the disjointed, mesenchymal state of the GS subtype.

## Defining the switch: epithelial-mesenchymal plasticity as a survival mechanism

### Reframing EMT not as a binary shift, but as a hybrid survival state

Traditionally viewed as a binary transition, the concept of EMT in GC has evolved into a spectrum of “Epithelial-Mesenchymal Plasticity (EMP)“ [45, 46]. Malignant gastric epithelial cells frequently enter a “partial EMT” or “hybrid E/M” state, rather than completing a full transition to a mesenchymal phenotype [47]. This intermediate state is not merely a transitional phase; it is a stable, adaptive “survival mode” in which cells co-express both epithelial (e.g., E-cadherin) and mesenchymal (e.g., vimentin) markers. Importantly, recent experimental evidence from single-cell RNA sequencing (scRNA-seq) investigations has demonstrated that this hybrid subpopulation possesses the greatest tumorigenic potential [46, 48], effectively functioning as the operational unit of the “Survival Switch” proposed in this review.

It is imperative to note that the ‘Survival Switch’ paradigm is not universally applicable to all GC subtypes. Although *H. pylori* is a universal risk factor, the GS subtype, which clinically corresponds to the diffuse-type histology, is the one in which the maintenance of the hybrid E/M state is most critical. The GS subtype is distinguished by a lack of mutations, but an enrichment of cell adhesion and motility pathways (e.g., Ras Homologue Family Member A (RHOA) mutations, Claudin-18 (CLDN18) fusions), in contrast to the CIN subtype, which is influenced by somatic copy number alterations. We hypothesize that the ‘Survival Switch’ serves as a critical “Pathogenic Divergence Point” in the gastric carcinogenesis landscape. The sequential, cohesive progression of intestinal-type GC (Correa’s cascade) is typically driven by chronic inflammation. However, the hyper-activation of the epigenetic ‘Lock’ (Phase II) compels cells to abandon this epithelial differentiation trajectory. These cells endure a lineage deviation as a result of the intense CagA signaling and METTL3-mediated locking, rather than progressing to cohesive intestinal metaplasia. They essentially “break out” of the Correa cascade, irrevocably losing E-cadherin and acquiring the high-motility, poorly cohesive features unique to the GS subtype. Consequently, the “Survival Switch” elucidates the mechanistic transition from a frequent bacterial stimulus to the specific, lethal biology of diffuse-type GC. Consequently, the therapeutic strategies that are proposed here are specifically designed for this ‘hardest-to-treat’ subgroup, as opposed to the MSI or EBV-positive subtypes, where immunotherapy may have a more prevalent impact.

### Linking the hybrid state directly to stemness and resistance

**Fig. 2:**
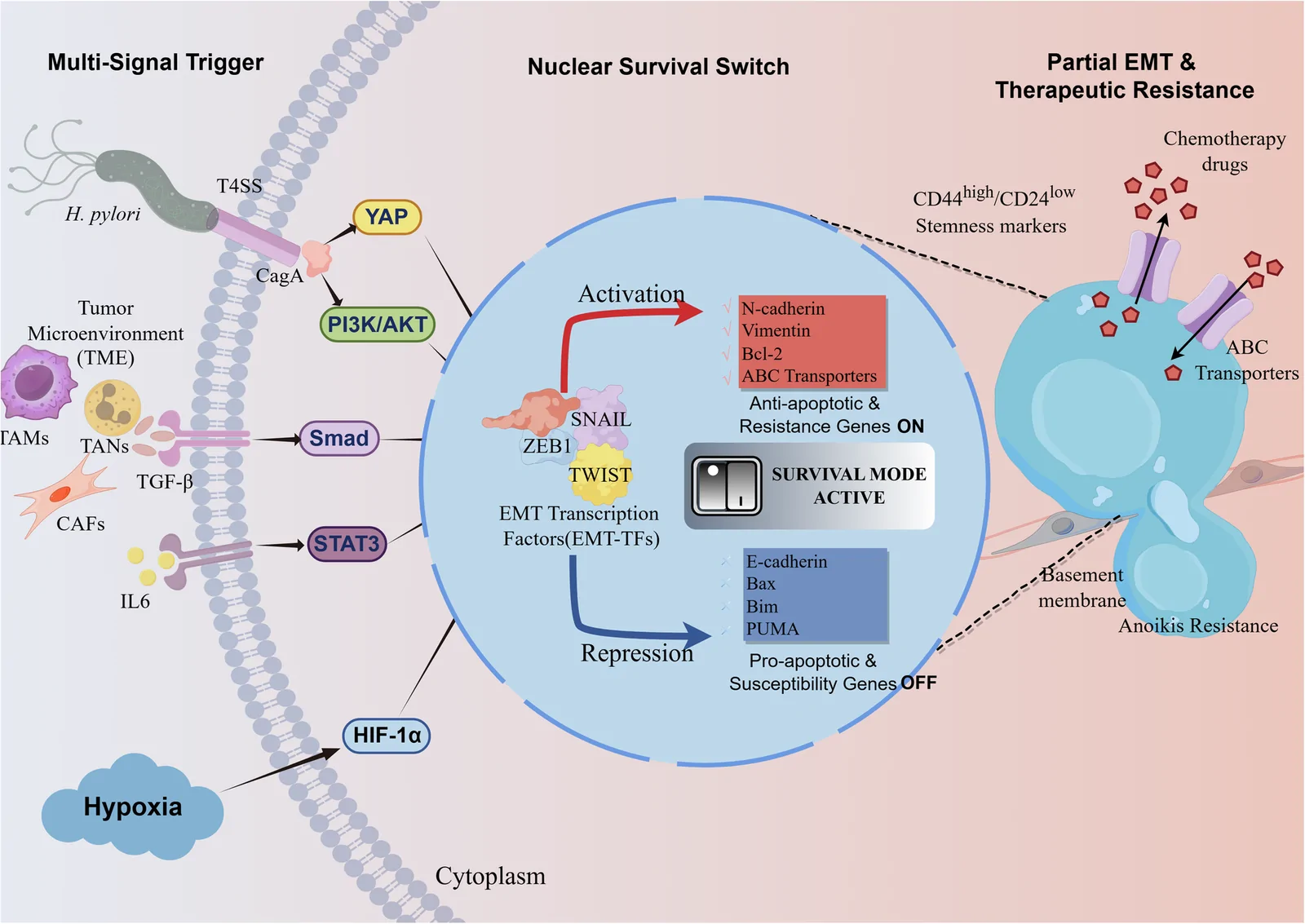
The EMT signaling network: a molecular switch for survival and drug resistance. This diagram demonstrates the manner in which the tumor microenvironment (TME) and *H. pylori* infection collaborate to decouple cell survival from proliferation. (Left) Multi-Signal Integration: The principal igniter is the virulence factor CagA, which is injected via the T4SS and activates the YAP and PI3K/AKT pathways [45, 60]. Simultaneously, inflammatory cytokines (Interleukin-6 (IL-6), Tumor Necrosis Factor-alpha (TNF-α), Transforming Growth Factor-beta (TGF-β)) from TAMs and CAFs, along with Hypoxia (Hypoxia-inducible factor-1 alpha (HIF-1α)), provide a sustained extrinsic drive [100, 102, 103]. (Center) The Nuclear Survival Switch: These pathways converge on essential EMT transcription factors (Zinc Finger E-box Binding Homeobox 1 (ZEB1), Snail Family Transcriptional Repressor 1 (SNAIL), Twist Family BHLH Transcription Factor 1 (TWIST)). This complex functions as a binary switch, inhibiting the transcription of pro-apoptotic genes (e.g., Bcl-2-associated X protein (Bax), Bcl-2-like protein 11 (Bim), p53 upregulated modulator of apoptosis (PUMA)) and epithelial markers (E-cadherin) while simultaneously upregulating anti-apoptotic shields (Bcl-2) and drug efflux pumps (ABC Transporters) [110, 151]. (Right) Hybrid Phenotype & Resistance: The output is a “Hybrid E/M” cancer stem cell (CSC) state (CD44^high^/CD24^low)^ characterized by anoikis resistance, dormancy, and intrinsic insensitivity to cytotoxic chemotherapy [37, 152].

The intrinsic relationship between cancer stem cell (CSC) properties and therapeutic resistance is the source of the clinical danger associated with this hybrid state. In order to circumvent cytotoxic death, cells in this plastic state activate anti-apoptotic pathways (e.g., B-cell lymphoma 2 (Bcl-2) upregulation) and drug efflux pumps (ATP-binding cassette (ABC) transporters) while simultaneously acquiring enhanced motility for dissemination (Fig. 2). This results in a fatal paradox: these quiescent, stem-like hybrid cells survive, forming a reservoir for recurrence, despite the fact that chemotherapy targets rapidly dividing epithelial cells. Additionally, the reversibility of this process—mesenchymal-epithelial transition (MET)—enables these disseminated cells to “switch off” the migratory program and re-acquire epithelial proliferative capacity for metastatic colonization at distal sites. Consequently, EMP functions as a dynamic toggle, facilitating the trade-off between invasion (EMT) and colonization (MET) in order to optimize survival in hostile environments [49–51]. The initial trigger that destabilizes the epithelial integrity and shifts this survival switch is often extrinsic, despite the fact that intrinsic cellular machinery sustains this plasticity. *H. pylori* serves as the primary catalyst for this cascade in GC.

## Phase I—the igniter: *H. pylori* CagA and the “Hit-and-Run” initiation

### Dismantling the barrier: junctional sabotage and loss of polarity

The Cag PAI is a significant virulence factor of *H. pylori*. It contains the genes for the effector protein CagA and a type IV secretion system (T4SS) [52]. CagA, the product of Cag PAI, is absorbed by gastric epithelial cells through the T4SS. CagA is initially released into the gastric epithelial cell by *H. pylori*, and it subsequently interacts with phosphatidylserine in host cells [53].

The phosphorylation of tyrosine residues within the EPIYA motifs of Abelson murine leukemia viral oncogene homolog 1 (Abl) and Steroid receptor coactivator (Src) kinases leads to an EMT-like phenotype following translocation. In vivo experimental models have demonstrated that *H. pylori*-infected mice develop GC in a CagA-dependent manner within a brief period of time, as demonstrated by research [54, 55].

**Fig. 3:**
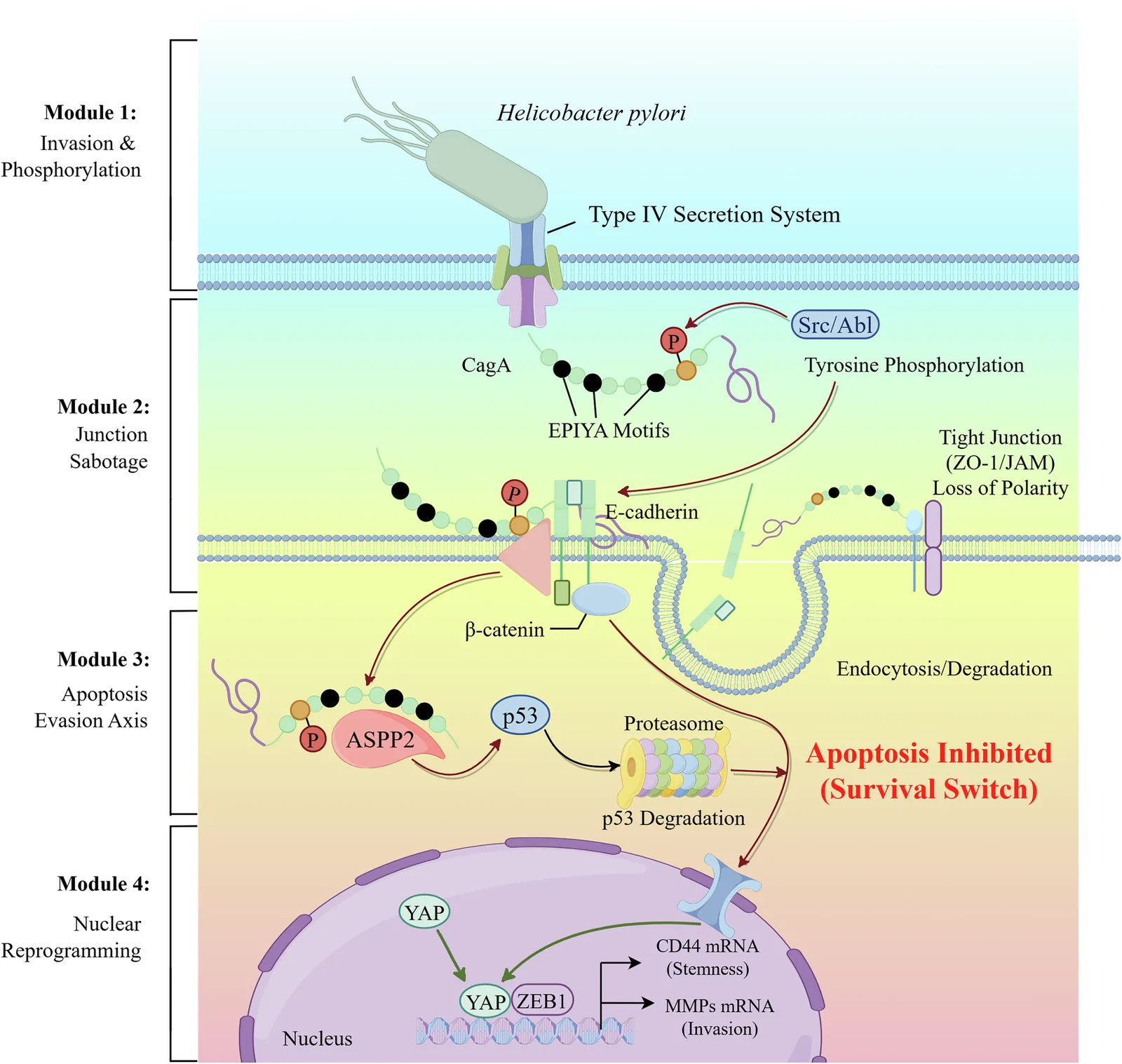
The CagA signalosome: hijacking polarity and p53 for apoptosis evasion and EMT. The multi-modular mechanism by which the *H. pylori* virulence factor CagA functions as the primary “igniter” of the survival switch is illustrated in this diagram. Junctional Sabotage and Polarity Loss: Module 1 and 2 (Top Panel). CagA is translocated into host cells through the Type IV Secretion System (T4SS) and subsequently phosphorylated at EPIYA motifs by Src/Abl kinases [52, 53]. The E-cadherin/β-catenin complex and tight junctions (ZO-1/JAM) are directly disrupted by phosphorylated CagA, resulting in the collapse of epithelial polarity and cytoskeletal reorganization necessary for motility [56, 58, 61]. (Middle Panel) Module 3: The Apoptosis Evasion Axis. CagA binds to the tumor suppressor ASPP2 to guarantee cell survival during this duress. This interaction not only inhibits ASPP2’s apoptotic function but also facilitates the proteasomal degradation of p53, thereby obliterating the cell’s intrinsic death mechanism (the “Survival Switch”) [61, 67]. (Bottom Panel) Module 4: Nuclear Reprogramming. The signalosome ultimately culminates in the nucleus, where CagA-driven signaling activates the YAP oncogenic pathway and upregulates the EMT-inducing transcription factor ZEB1 [64, 101]. This transcriptional shift drives the expression of invasion mediators (e.g., MMPs) [58] and stemness markers (e.g., CD44), establishing the drug-resistant hybrid phenotype [151].

An intricate network of molecular interactions is involved in the transition from CagA translocation to EMT activation. The disruption of the E-cadherin/β-catenin adhesion complex is a critical step. By directly disrupting this complex, CagA induces the nuclear translocation of β-catenin, thereby’sabotaging’ the cell’s structural integrity (Fig. 3). Cellular activity is altered by the elimination or obliteration of E-cadherin, a fundamental epithelial marker [56, 57].

Additionally, through formation of actin pseudopodia, CagA can enhance ECM degradation, stimulate cell motility and invasion, and trigger Matrix Metalloproteinase-7 (MMP-7) expression [58–60]. Additionally, CagA disrupts the interaction between the PAR complex and the apoptosis-stimulating protein of p53 (ASPP2), thereby altering cell polarity structures. The subsequent nuclear reprogramming is contingent upon the physical dismantling of polarity [61].

It is crucial to recognize that the term “Hit-and-Run” does not necessarily indicate a solitary, transient contact. Rather, the ‘Igniter’ phase necessitates a certain level of chronicity. The accumulation of inflammatory stimuli is essential for the breach of the epithelial threshold, despite the fact that CagA is the primary molecular spark. The pro-inflammatory milieu that destabilizes cell junctions is mediated by the persistent activation of the NF-κB pathway by bacterial peptidoglycans and the virulence factor VacA, even in CagA-negative strains. Nevertheless, CagA functions as a designated “accelerator” that significantly reduces the time necessary to reach a theoretical “Point of No Return.” Therefore, we conceptually frame the ‘Igniter’ as not merely a molecular event; it is a temporal process in which the cell’s capacity for restitution is eventually overcome by cumulative epigenetic noise, thereby activating the survival switch.

### Nuclear reprogramming: hijacking YAP/ZEB1 and evasion of apoptosis

CagA initiates a transcriptional overhaul upon the physical barrier being breached. CagA directly orchestrates a transcriptional remodeling by hijacking the PI3K-Akt/Wnt axis to upregulate ZEB1 and subverting the Hippo tumor suppressor pathway to activate the oncogenic YAP co-activator upon breaching the physical barrier [62–65]. The long-term EMT program is initiated by the direct expression of invasion mediators, which is driven by this dual commandeering [65]. Furthermore, the activation of matrix metalloproteinase-3 (MMP-3) is facilitated by EPIYA-C-phosphorylated CagA, which in turn promotes a long-term EMT program [66].

The evasion of cell demise is essential for the ‘Survival Switch’. In addition to disrupting polarity, the interference with ASPP2 previously mentioned has a dual effect: it simultaneously promotes p53 degradation and impairs ASPP2’s pro-apoptotic activity, thereby suppressing apoptosis. CagA effectively dismantles the epithelial integrity and suppresses early apoptotic barriers through these mechanisms [67].

Nevertheless, the physical presence of the bacterium is a determining factor in these direct molecular interactions. They do not provide an explanation for the clinical paradox: why does gastric carcinogenesis persist even after *H. pylori* has been effectively eradicated? This implies that CagA does not merely disrupt existing cellular states; rather, it activates a self-sustaining mechanism that endures the infection.

## Phase II—The lock: epigenetic scarring and the “point of no return”

Clinical evidence indicates that the risk of cancer persists in a subset of patients even after successful bacterial clearance, despite the fact that current guidelines prioritize the eradication of *H. pylori* as the primary prevention for GC. The most adequate explanation for this paradox is the “Hit-and-Run” mechanism [68, 69].

Although this review emphasizes epigenetic mechanisms, post-eradication persistence is undoubtedly multifactorial. There are numerous alternative and complementary explanations that must be taken into account. Initially, the pre-existing clonal selection hypothesis posits that a subset of genetically advanced sub-clones (e.g., those that contain TP53 or RHOA mutations) have already arisen by the time *H. pylori* is eradicated. These clones are endowed with intrinsic survival advantages and will persist in their expansion regardless of the infection [70, 71]. Secondly, the concept of stromal dominance posits that the tumor microenvironment (TME) surrounding the tumor is permanently corrupted. CAFs and TAMs assume the role of the primary driver, autonomously secreting factors (such as TGF-β and IL-6) that maintain malignant progression without the need for bacterial stimulation [72, 73]. Lastly, clinical interventions can lead to treatment-induced selection, in which subsequent therapies inadvertently function as evolutionary bottlenecks, enriching the most robust, treatment-resistant mesenchymal cells [74]. In conjunction with these mechanisms, we suggest that the ‘Hit-and-Run’ epigenetic scarring functions as a critical intracellular safeguard.

Based on these clinical observations, we hypothesize that CagA induces enduring “epigenetic scarring” that effectively “locks” gastric epithelial cells in a partial-EMT state. This scarring includes DNA methylation of Cadherin-1 (CDH1) and, most importantly, METTL3-mediated m^6^A modifications [75–78].

Based on this epigenetic remodeling, we propose a theoretical model for a molecular ‘Point of No Return.’ At this hypothetical threshold, CagA-induced EMT transitions from a transient, pathogen-dependent response to a highly stable, self-sustaining, and autonomous epigenetically fixed driver [79]. While this ‘Point of No Return’ remains a conceptual hypothesis, indirect experimental support is provided by recent scRNA-seq studies, which have identified a distinctive subpopulation of “hybrid gastric-intestinal” cells in Incomplete Intestinal Metaplasia (Inc-IM) that resembles this partial-EMT state [80]. Once this hybrid population is established, the survival switch becomes autonomous.

### The epigenetic lock: METTL3 in the context of global epigenetic regulation

The transient signaling initiated by bacteria must be consolidated into a stable cellular memory once this proposed “Point of No Return” is crossed. Epigenetic regulators are responsible for this maintenance. METTL3 serves as the principal stabilizer among these. Preclinical studies have established that the invasive capability of GC cells was conferred by the increase in methyltransferase-like 3 (METTL3) in GC. ZMYM1 mRNA expression is significantly increased by METTL3 through an m^6^A-HuR signaling pathway, which subsequently inhibits E-cadherin transcription by recruiting the CtBP/LSD1/CoREST complex. METTL3 cultivates a tumorigenic environment that facilitates immune evasion, enhances cell proliferation, and confers resistance to pharmacological therapies through these mechanisms [81, 82]. Song et al. discovered that the homeobox gene A10 (HOXA10), a transcription factor, activates the Smad signaling pathway by upregulating transforming growth factor β2 (TGFB2). METTL3 expression is additionally stimulated by activated Smad signaling. Therefore, the TGFB2/Smad/METTL3 signaling axis may be activated by HOXA10, thereby inducing EMT [83]. Yuan and colleagues described a substantial correlation between p-Smad3 and METTL3 in a variety of cancer types. In the TGF-β/Smad signaling pathway, p-Smad3 is a critical transcription factor that directly interacts with promoter regions of target genes to regulate their expression [84].

This bidirectional interaction implies that the TGF-β/METTL3 axis functions as a molecular “latching” mechanism, rather than merely a linear pathway. The positive feed-forward loop is established by the initial induction of METTL3 by TGF-β, which in turn promotes further Smad activation. The conceptual ‘point of no return’ in our proposed survival switch is theoretically elucidated by this mechanism: the EMT program becomes independent of the initial bacterial driver, thereby locking cells in a drug-resistant state, once this epigenetic loop is fully activated.

Although METTL3-mediated m6A modification serves as a critical rapid-response ‘writer’ that stabilizes EMT-TFs post-transcriptionally, the persistent ‘locking’ of the mesenchymal state requires a synergistic interaction with global chromatin remodelers. In order to gain a more comprehensive understanding of the relative significance of METTL3, it is necessary to compare it to other critical epigenetic regulators, including Enhancer of Zeste Homolog 2 (EZH2), DNA Methyltransferase 1 (DNMT1), and Bromodomain-containing Protein 4 (BRD4) [85].

EZH2 (the catalytic core of the Polycomb Repressive Complex 2, PRC2) ensures a repressive chromatin state that is exceedingly stable, in contrast to the dynamic and reversible RNA regulation of METTL3 [86–88]. Epithelial barriers, including CDH1 (E-cadherin), are frequently silenced by METTL3-driven complexes (such as C-terminal Binding Protein (CtBP)/ Lysine-specific Demethylase 1 (LSD1)), but they are ultimately consolidated by EZH2 through the deposition of H3K27me3 marks. As a result, maintenance DNA methyltransferases, specifically DNMT1, are recruited to hypermethylate promoter 5’-Cytosine-phosphate-Guanine-3’ (CpG) islands, thereby establishing the ultimate long-term cellular memory that “locks” the scar in place [89, 90].

In contrast, epigenetic “readers” such as BRD4 function as potent co-activators, whereas EZH2 and DNMT1 mediate silencing. In order to initiate the sustained hyper-transcription of mesenchymal oncogenes (e.g., c-Myc, ZEB1) that *H. pylori* initially triggered, BRD4 recognizes acetylated histones [91, 92].

Consequently, in this epigenetic hierarchy, METTL3 serves as the critical translational “bridge” that responds to acute TME stress, while EZH2, DNMT1, and BRD4 establish a robust structural foundation that permanently stabilizes the chromatin in a therapy-resistant configuration [93].

### Classical signaling cascades and ncRNA interactions

Classical signaling cascades serve as the amplifier to maintain high-grade plasticity, while epigenetic scarring provides the foundational memory. These molecular amplifiers can be widely classified into kinase cascades (e.g., PI3K/AKT) that provide rapid signal transduction and non-coding RNAs that offer fine-tuned post-transcriptional regulation. The complete establishment of EMT necessitates the amplification of this signal by host pathways [94]. The PI3K/AKT/mTOR axis acts as a critical bridge here [95]. It is not only directly recruited by CagA’s EPIYA motifs but also forms a self-sustaining loop that increases the levels of ZEB1, SNAIL, and Slug well beyond the initial bacterial stimulus. This guarantees that the’survival switch’ remains operational regardless of fluctuations in bacterial burden.

**Fig. 4:**
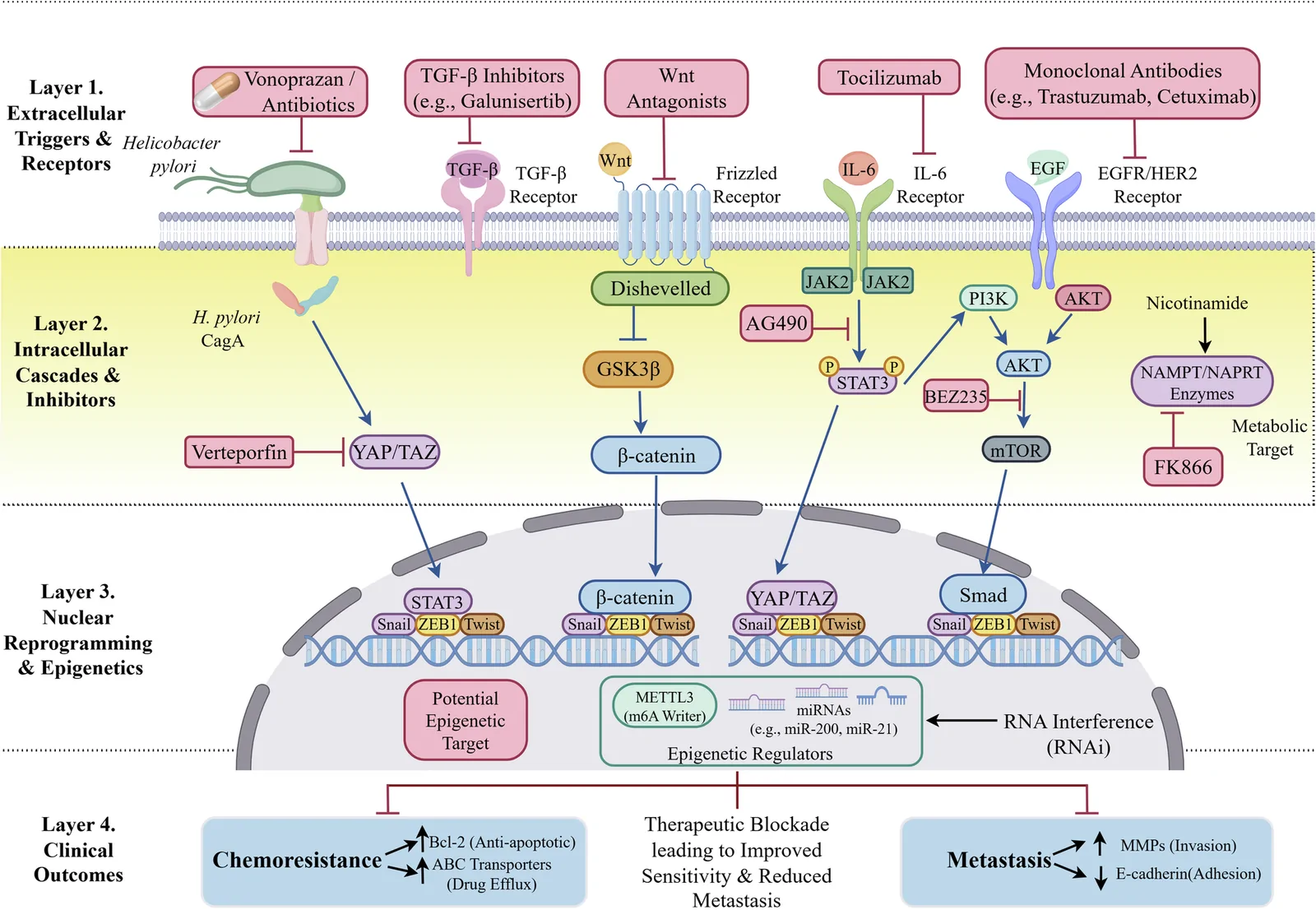
Targeting the EMT signalosome: therapeutic vulnerabilities and interventions in GC. This diagram illustrates the multi-layered signaling network driving EMT and potential therapeutic interventions. (Layer 1 & 2) Extracellular Triggers and Intracellular Cascades: The *H. pylori* virulence factor CagA translocates via T4SS to activate the YAP/TAZ axis [64, 101, 151]. TGF-β binds its receptor to activate Smad complexes [81, 83, 153]; EGF and HER2 receptors initiate the PI3K/AKT/mTOR cascade [154–156]; Wnt signaling inhibits GSK3β via Frizzled receptors to stabilize β-catenin [157, 158]; and IL-6 transduces signals through the JAK2/STAT3 pathway [103, 159]. Specific inhibitors targeting these nodes (e.g., Verteporfin, Galunisertib, AG490, BEZ235) are highlighted in red boxes. (Layer 3) Nuclear Reprogramming and Epigenetics: These pathways converge in the nucleus to activate core EMT transcription factors (Snail, ZEB1, Twist). At this stage, METTL3-mediated m^6^A modification acts as an “epigenetic lock” to maintain the mesenchymal phenotype [81, 82]. (Layer 4) Clinical Outcomes: The activation of this network drives chemoresistance via the upregulation of Bcl-2 and ABC transporters [110], and promotes metastasis through MMPs upregulation and E-cadherin suppression [58–60, 125].

Furthermore, the intrinsic cellular apparatus functions as a signal amplifier and is significantly influenced by non-coding RNAs (e.g., specific miRNAs and circRNAs). These host factors compete to modulate classical cascades, such as hyperactivating the PI3K/AKT/mTOR axis or sensitizing the TGF-β/Smad and Wnt/β-catenin pathways, rather than operating in isolation (Fig. 4). Despite the absence of a continuous *H. pylori* stimulus, this complex cross-talk guarantees that the nuclear concentration of EMT-TFs (ZEB1, SNAIL) remains above the threshold necessary to maintain the ‘epigenetic lock' [96]. TGF-β could be a triggering factor for the initiation of the mesenchymal subtype GC [97]. The complex interplay of these signaling cascades is illustrated in Fig. 4.

## Phase III—the niche: TME-driven reinforcement and plasticity maintenance

**Fig. 5:**
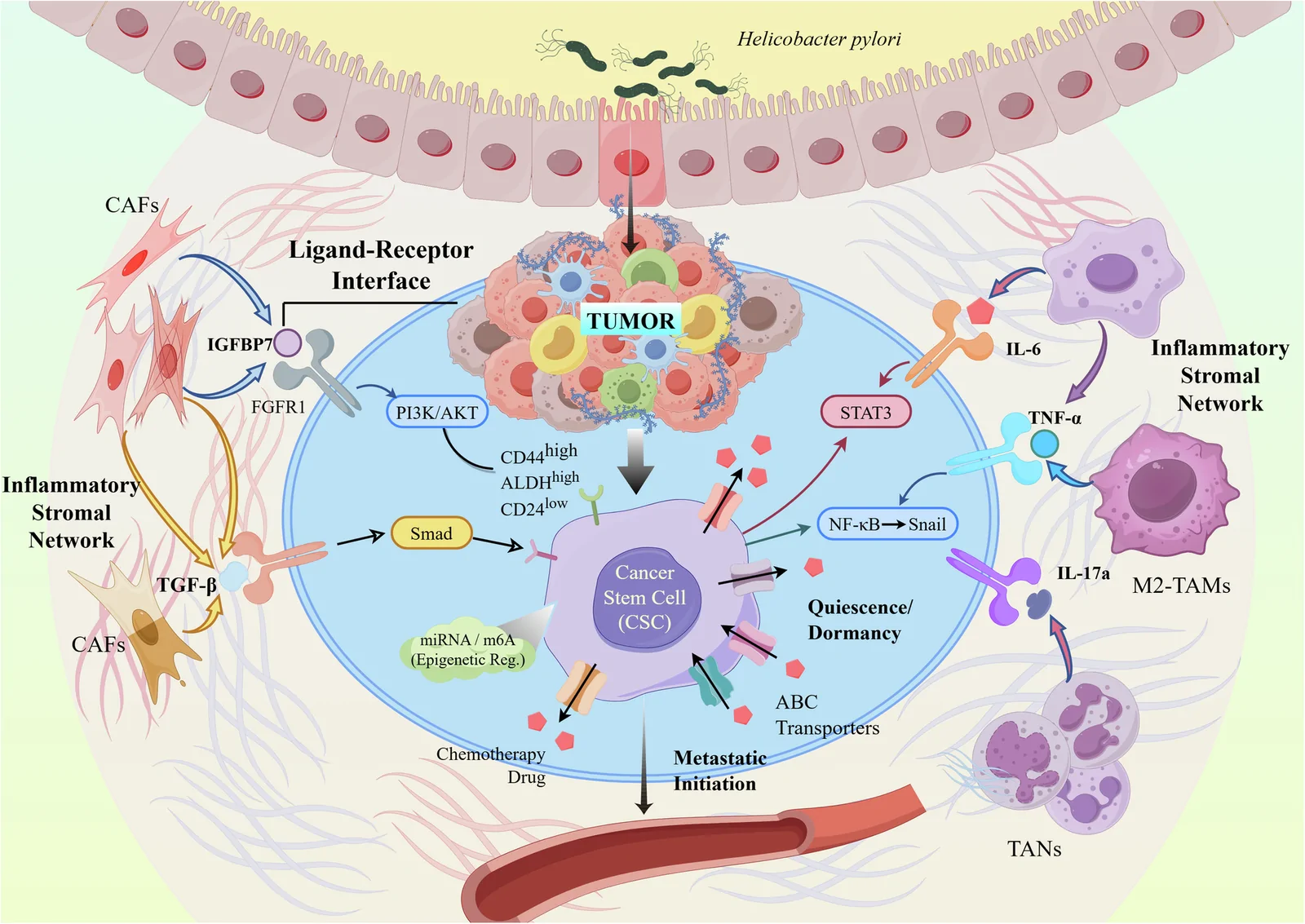
The inflammatory ecosystem: extrinsic reinforcement of plasticity and the emergence of drug-resistant CSCs. The mesenchymal phenotype is consolidated by the tumor microenvironment (TME) independently of the initial *H. pylori* trigger, as illustrated in this schematic, which demonstrates the “amplification” mechanism. (Left) Stromal Reinforcement by CAFs: Cancer-Associated Fibroblasts (CAFs) transform the extracellular matrix and secrete TGF-β, which induces EMT through Smad and PI3K signaling [100]. Additionally, CAFs secrete IGFBP7, which binds to FGFR1 on tumor cells to activate the PI3K/AKT axis, reinforcing the stem-like phenotype [105]. (Right) The Immune Inflammatory Niche: A symbiotic network of M2-Tumor-Associated Macrophages (TAMs) and Tumor-Associated Neutrophils (TANs) sustains plasticity. TAMs release IL-6 to activate STAT3 [102, 103] and TNF-α to stabilize Snail via the NF-κB pathway [104]. TANs secrete IL-17a, providing a convergent signal to amplify STAT3 activation [107]. (Center) The Drug-Resistant CSC Core: These converging extrinsic signals enshrine cancer cells in a dormant, “hybrid E/M” Cancer Stem Cell (CSC) state (CD44^high^/CD24^low^). This state is distinguished by the upregulation of ABC transporters, metabolic quiescence, and epigenetic reprogramming (miRNA/m^6^A), which leads to intrinsic chemotherapy resistance and metastatic initiation [109, 110, 152].

The convergence of intrinsic and extrinsic forces is essential for the establishment of the ‘Survival Switch’. Although *H. pylori* initiates this lethal state through its ‘Hit-and-Run’ mechanism, the state’s long-term persistence necessitates more than just epigenetic memory. This shift represents a state of stromal dominance. The corrupted stroma does not merely replace the pathogen after bacterial eradication; rather, it acts as an autonomous driver, establishing a ‘resonance chamber’ that independently maintains and enhances the intracellular epigenetic program. As demonstrated in Fig. 5, this inflammatory ecosystem, which includes CAFs, TAMs, and TANs, generates a dual-lock system that protects the mesenchymal state from spontaneous reversion (MET) by providing a compulsory extrinsic signal.

### Cancer-associated fibroblasts (CAFs): the architects of invasion

The behavior of cancer cells is orchestrated by stromal constituents, with CAFs acting as key drivers of EMT [98]. CAFs promote malignancy by secreting proteases like MMPs to remodel the extracellular matrix (ECM) and cytokines such as TGF-β [99]. TGF-β signaling, heightened by CAF infiltration, activates Smad complexes and MAPK/PI3K pathways to trigger EMT, while also upregulating TCF4 via miR-1306/33a modulation [100]. Crucially, this paracrine signaling provides a ‘third hit’ to the EMT machinery [100]. ZEB1, already upregulated intracellularly by *H. pylori* and amplified by host signaling loops, is further reinforced extracellularly by CAFs [100, 101]. CAFs effectively establish an external “niche” that reinforces the epigenetic lock by directly inducing ZEB1 transcription. This extrinsic support functions as a structural buttress, guaranteeing that the mesenchymal state is preserved even when intrinsic signaling temporarily weakens, thereby rendering therapeutic reversal significantly more difficult [100].

### Tumor-associated macrophages (TAMs): the M2 polarization axis

The mesenchymal phenotype is reinforced by the symbiotic relationship between GC cells and TAMs, which are primarily of the M2 subtype. A multifaceted cytokine network facilitates this interaction:

I. The IL-6/STAT3 Axis: GC-derived mesenchymal stromal cells (GC-MSCs) secrete IL-6 and IL-8, which persistently activate the STAT3 signaling hub in tumor cells to reinforce the expression of mesenchymal markers (e.g., Vimentin) [102]. Moreover, the overexpression of RHOJ GTPase has been found to amplify this IL-6 signaling loop [103].
II. The TNF-α and TGF-β Loops: TAMs secrete TNF-α, which stabilizes Snail through NF-κB activation, and TGF-β, which preserves mesenchymal characteristics. The transcription factors that CagA initially commandeered are the same set upon which this inflammatory cytokine network converges. For instance, although CagA degrades p53 to evade apoptosis (Section 3), TAM-secreted TNF-α stabilizes Snail via NF-κB to obtain a comparable survival advantage [104]. This convergence of intrinsic (bacterial) and extrinsic (immune) signals on the Snail/ZEB axis creates a robust, redundant network that sustains stemness and drug resistance.
III. The Insulin-like Growth Factor Binding Protein 7 (IGFBP7)-FGFR1 Signaling: Recent RNA-seq data reveals that TGF-β from TAMs upregulates IGFBP7, which in turn activates the FGFR1/PI3K/AKT pathway, creating a feed-forward loop that correlates with poor patient outcomes [105]. Additionally, the serine protease PRSS23 has been shown to regulate macrophage infiltration by modulating FGF2 secretion, further highlighting the plasticity of this immune niche [106].

### The stress niche: neutrophils and hypoxia

Tumor-associated neutrophils (TANs) accumulate at the invasion margin in addition to macrophages. They secrete IL-17a to activate the JAK2/STAT3 pathway, which further suppresses E-cadherin and promotes metastasis (Fig. 5) [107].

Lastly, the “survival switch” is triggered by the physical microenvironment, particularly hypoxia, which functions as a universal stressor. E-cadherin is transcriptionally repressed by hypoxia-inducible factor 1α (HIF-1α), which increases the levels of ZEB and TCF. This forces cells to transition to a mesenchymal state in order to escape the oxygen-deprived core. Environmental stress is directly linked to plasticity by the fact that this hypoxic niche frequently overlaps with regions of stem cell maintenance [108].

## The clinical stalemate: why standard therapies fail against the hybrid state

Notwithstanding progress in surgical and systemic treatments, the outlook for advanced stomach cancer remains bleak. The primary reason for this failure is not solely the tumor’s aggressiveness, but a basic mechanistic discord between our therapeutic options and the biological condition of EMT-transformed cells.

### The target mismatch: targeting proliferation in a quiescent core

The premise of standard-of-care regimens, such as fluoropyrimidines and platinum agents, is to target swiftly dividing cells. Nevertheless, the “Survival Switch” is activated, which effectively separates survival from proliferation. Cytotoxic agents that target DNA replication are rendered metabolically invisible to cells in the hybrid E/M state, as they frequently enter a slow-cycling or quiescent phase (dormancy) [109]. Concurrently, these cells enhance the activation of defense mechanisms. As previously mentioned, the EMT program directly regulates the expression of ABC transporters for drug efflux and anti-apoptotic proteins (e.g., Bcl-2), thereby establishing a multilayered defense against apoptotic stimuli [110]. As a result, conventional chemotherapy functions as a rudimentary filter, effectively debulking the proliferative epithelial mass while leaving the resistant, stem-like mesenchymal core unaffected.

### Iatrogenic selection: when treatment reinforces the switch

Importantly, clinical interventions may inadvertently reinforce the apparatus they are designed to destroy. The “Survival Switch” is susceptible to environmental duress. There is evidence to imply that exposure to sublethal concentrations of chemotherapeutics (e.g., 5-FU) acts as a stress signal that further activates EMT pathways (TGF-β, Wnt) [111, 112]. This “iatrogenic selection” elucidates the clinical phenomenon of rapid recurrence with enhanced metastatic potential following an initial response. Through this treatment-induced clonal selection, the therapy itself converts a heterogeneous tumor into a refractory mesenchymal dominance by selecting for the most plastic, drug-resistant sub-clones [112–114].

This results in a therapeutic paradox: we are unable to eliminate these cells due to their dormancy (the barrier), but we cannot merely leave them alone because they are the precursors to relapse. Consequently, in order to eliminate this resistant reservoir, we must first compel it to depart this protected state, thereby reactivating it and rendering it vulnerable once more.

## Reprogramming the network: from “search and destroy” to “wake-up and kill”

Radical surgery and perioperative adjuvant chemoradiotherapy have been the primary therapies for resectable GC. In the past, the conventional approach to advanced GC has been to combine immunotherapy (PD-1 antibody: pembrolizumab/nivolumab, PD-L1 antibody: adebrelimab), targeted therapy (HER2/neu or ERBB2: trastuzumab, VEGFR2: ramucirumab/rivoceranib), and cytotoxic chemotherapy (fluoropyrimidine, platinum) [2, 115, 116]. However, we have determined that the epithelial-to-mesenchymal transition is a prevalent occurrence in GC, which offers a novel approach to targeted treatment.

Three primary strategies can be employed for targeted EMT therapy, as previously mentioned: targeting cancer cells and ECM, inhibiting upstream signaling pathways, and targeting quasi-mesenchymal (qM) cells and ECM [117]. In terms of both clinical and conceptual benefits, patients may benefit from the reversal of EMT and the production of MET. In essence, this method compels cancer cells that are in a stem-like state during the execution of the EMT program to transition into non-stem cell types and reestablish their epithelial characteristics. Consequently, these cells lose their resistance to specific medicinal interventions and their enhanced capacity to develop malignancies [118].

### Targeting epigenetic and molecular drivers

Targeting the METTL3/ZMYM1 axis, which we identified as a critical epigenetic driver of EMT in Section 4.1, is a promising therapeutic frontier. In contrast to strategies that indiscriminately inhibit methylation, those that concentrate on disrupting the specific recruitment of the CtBP/LSD1/CoREST complex provide improved precision. Preclinical evidence indicates that the suppression of E-cadherin induced by ZMYM1 can be effectively counteracted by the attenuation of these complex components (CtBP1, LSD1, or CoREST1) [81]. This implies that unlatching this particular epigenetic “lock” can reverse the mesenchymal phenotype and reestablish chemosensitivity, thereby validating the axis as a viable pharmacological target for biomarker-guided therapy.

In contrast, classical epigenetic therapies that target DNA and histone modifications, such as DNMT1 inhibitors like Decitabine, EZH2 inhibitors like Tazemetostat, and BET/BRD4 inhibitors, have demonstrated varying degrees of efficacy in reversing EMT in solid tumors. However, they frequently suffer from broad, non-specific toxicity. The therapeutic advantage of targeting the METTL3 axis is its position upstream of permanent chromatin remodeling. METTL3 inhibition may provide a more dynamic and specific approach to “unlock” the Survival Switch before the cells are irreversibly entrenched by DNMT1 and EZH2 by intercepting the rapid translational stabilization of EMT-TFs [93, 119, 120].

### Targeting the ‘ghost’: overcoming the Hit-and-Run legacy

While *H. pylori* eradication remains the cornerstone of GC prevention, its efficacy diminishes once the EMT “survival switch” has been fully activated [121, 122]. Current recommendations emphasize eradication regimens, including Vonoprazan-based triple therapy, which have demonstrated superior eradication rates in comparison to conventional therapies [123]. Nevertheless, this approach is predominantly focused on the disease’s originator and may not be able to reverse the established malignancy. The virulence factor CagA has the potential to trigger self-sustaining epigenetic lesions and molecular cascades that persist even after the bacterium is eliminated, as previously discussed in the “Hit-and-Run” paradigm [124]. Therefore, a clinical disconnect exists: removing the pathogen does not necessarily “unlock” the EMT state in refractory tumors.

Subsequently, clinical intervention must progress beyond mere eradication. We suggest a therapeutic approach that is stratified: Although a ‘dual-intervention’ approach is indeed necessary for *H. pylori*-positive patients (eradication to prevent new insults and inhibitors to block signaling), the ‘Hit-and-Run’ nature of CagA necessitates consideration of patients with history of infection or established ‘epigenetic scarring’. Eradication alone is insufficient for this subgroup, which is identifiable by elevated mesenchymal signatures (e.g., MEMTS). Rather, clinical intervention must address the “Ghost” architecture from two perspectives: the epigenetic scar and the signaling pathways that perpetuate it. In order to “starve” the phantom of its maintenance cues, signaling inhibitors (such as Verteporfin or PI3K inhibitors) are essential agents, as the epigenetic lock (mediated by METTL3 and methylation) depends on dynamic kinase cascades to maintain its stability (as described in Section 4.2). It is imperative that these be combined with epigenetic remodelers (e.g., METTL3 inhibitors or demethylating agents) to directly eliminate the molecular memory in order to achieve a complete reset. This “Dual-Strike” strategy effectively dismantles the autonomous EMT apparatus, treating the molecular “ghost” regardless of the current infection status [121, 124].

### Normalizing the immune niche: neutrophils and macrophages

In GC, tumor-associated neutrophils (TANs) facilitate tumor invasion and lymph node metastasis. Neutrophil concentrations are correlated with tumor stage and can predict an unfavorable prognosis. In the future, the frequency of intra-tumoral neutrophils may function as a significant clinical marker. TANs can enhance EM in GC by secreting interleukin-17a (IL-17a), which promotes the JAK2/STAT3 signaling pathway in GC cells. In GC cells, the specific JAK2/STAT3 pathway inhibitor AG490 and an IL-17a-neutralizing antibody can effectively reverse TAN-induced phenotypes. TGF-β1 was used by GC cells to direct neutrophils to increase the synthesis of FAM3C through Smad2/3 signaling. This subsequently enables EMT and lymph node metastasis (LNM) in GC through the JNK-ZEB1/Snail pathway. AB72182, which functions as a neutralizing antibody, has the ability to inhibit FAM3C in order to reverse the EMT pathway [125, 126].

### The proposed “wake-up and kill” strategy: exploiting the vulnerability window

However, targeting EMT is a double-edged sword. As noted, while reversing EMT (inducing MET) strips cancer cells of their stem-like drug resistance [127], it may inadvertently promote the colonization of circulating tumor cells (CTCs) by restoring their proliferative capacity at distant sites [128].

This risk is of the utmost importance; the ‘Wake-up’ phase could catastrophically accelerate the precise metastatic progression it is designed to prevent if the subsequent cytotoxic ‘Kill’ phase is not perfectly synchronized. Re-epithelialized cells regain robust cell-cell adhesion (e.g., E-cadherin expression) and proliferative capabilities, which are absolute prerequisites for macrometastasis formation in distal organs such as the liver or lungs. Thus, the clinical implementation of this approach is confronted with a significant pharmacokinetic obstacle: it necessitates the precise temporal coordination of the MET-inducing agent and the cytotoxic drug to reach the tumor niche, a task that is notoriously challenging to accomplish with systemic administration. This creates a therapeutic paradox: how to re-sensitize tumors without accelerating metastasis?

The concept of a “therapeutic vulnerability window” is essential for resolving this issue. The induction of MET must not be an isolated treatment; rather, it should be a component of a precisely timed “Wake-up and Kill” strategy, a conceptual therapeutic model we propose here. The objective is not to completely restore a colonization-competent epithelial phenotype, but rather to compel quiescent cells to enter a “Metabolic Trap.” The induction of MET induces the re-entry of dormant cells into the cell cycle, a process that results in severe oxidative stress (ROS accumulation) and metabolic exhaustion. This results in a transient “hyper-vulnerable window” in which cells have lost their mesenchymal drug resistance but have not yet re-established the robust cell-cell adhesion complexes necessary for successful colonization. We exploit their metabolic fragility before they can establish a foothold in distal organs by synchronizing high-intensity cytotoxic chemotherapy with this specific transitional phase [129, 130]. While supported by in vitro metabolic rationales, this synchronized approach requires rigorous validation through future randomized clinical trials. Therefore, clinical success of anti-EMT therapy hinges on precise sequencing rather than simple inhibition [131].

### Biomarker-guided patient stratification via liquid biopsy

The definition of a precise, multi-dimensional biomarker panel that indicates the activation of the ‘Survival Switch’ concept is essential for its successful translation into clinical oncology. A singular marker is insufficient due to the fact that the ‘Hybrid E/M’ state is a spectrum rather than a binary outcome [132, 133]. We suggest a ‘Activated Survival Switch Signature’ that consists of three distinct tiers: (1) The Epigenetic/Transcriptional Tier: Nuclear accumulation of core EMT-TFs (ZEB1, SNAIL) and overexpression of the m6A writer METTL3 and its specific downstream targets (e.g., ZMYM1) [134]; (2) The Phenotypic/Stemness Tier: The paradoxical co-expression of epithelial (EpCAM, E-cadherin) and mesenchymal (Vimentin, N-cadherin) surface markers, intrinsically linked to the CD44^high^/CD24^low^ cancer stem cell profile [132]; and (3) The TME Niche Tier: High spatial enrichment of stromal-derived maintenance factors, particularly CAF-secreted IGFBP7 and TAM-derived IL-6/TGF-β [135]. The initial stage in patient stratification is the identification of this composite signature through spatial transcriptomics or multiplex immunohistochemistry (mIHC) [136, 137].

However, while histological assessment of these aforementioned biomarkers in primary tumor tissues provides a static snapshot, it frequently fails to capture the spatiotemporal fluidity of the metastatic cascade or the post-treatment adaptive resistance [138, 139]. To bridge this gap, liquid biopsy offers a non-invasive avenue to monitor the dynamic status of the ‘survival switch’ in real time [140]. Peripheral blood can be used to distinguish circulating tumor cells (CTCs) that are undergoing EMT, specifically those that exhibit a “hybrid E/M” or wholly mesenchymal phenotype (e.g., Vimentin(+)/Cytokeratin(-) cells) [140, 141]. These EMT-transformed CTCs are not merely prognostic markers for poor survival but serve as direct indicators of therapeutic resistance.

In the clinical setting, the presence of Vimentin + /CK- CTCs is associated with intrinsic resistance to standard cytotoxic regimens, which are predominantly used to target rapidly proliferating epithelial cells. These regimens include 5-FU and platinum. Consequently, liquid biopsy screening could be a critical stratification tool: patients with a high burden of mesenchymal-CTCs may benefit from treatment with EMT-modulating compounds upfront (to induce MET) prior to cytotoxic chemotherapy [118, 142]. Additionally, longitudinal monitoring functions as tactical catalyst for the “Kill” phase. The objective is to identify the onset of phenotypic drift, specifically the emergence of “partial-MET” CTCs (co-expressing Vimentin and EpCAM), rather than waiting for a complete epithelial transition. The onset of the “Metabolic Vulnerability Window” as previously mentioned is indicated by the detection of this early transition. By intervening at this highly unstable intermediate stage, clinicians can mitigate the severe risk of promoting fully colonization-competent epithelial CTCs, thus safely executing the strategy without triggering explosive metastasis. This real-time intelligence enables clinicians to preemptively deploy cytotoxic agents, stopping the metastatic cascade before colonization competence is fully acquired by eliminating the “waking” cells while they are metabolically stressed and circulating [143].

### Emerging preclinical modalities: overcoming the delivery barrier

While small molecule inhibitors (SMIs) targeting upstream signals show promise, the direct modulation of EMT-driving transcription factors (EMT-TFs) remains the “holy grail” of preventing metastasis [144]. The miR-200 family of non-coding RNAs (ncRNAs) serves as natural “epithelial gatekeepers” by directly repressing the ZEB1/2 axis [145]. The theoretical reversal of the mesenchymal phenotype and the re-sensitization of GC cells to chemotherapy could be achieved by restoring these tumor-suppressive miRNAs. Nevertheless, the clinical implementation of RNA therapeutics has been historically impeded by substantial pharmacokinetic obstacles, including their rapid degradation by serum nucleases and inadequate intracellular penetration [146].

This is where nanotechnology offers a transformative solution, shifting its role from a mere cytotoxic agent to a sophisticated delivery vehicle [147]. Liposomes or polymeric nanoparticles are examples of advanced nanocarriers that can safeguard delicate genetic payloads, such as anti-EMT siRNA or miR-200 mimics, from degradation [148]. Additionally, nanoplatforms can ensure high-concentration delivery to the metastatic niche by surface-modifying with tumor-homing ligands or utilizing the enhanced permeability and retention (EPR) effect [149, 150]. This cutting-edge frontier of nucleotide-based precision medicine and nanotechnology has the potential to transform undruggable EMT targets into clinically viable realities, thereby overcoming the “delivery bottleneck”.

## Conclusions and outlook: from “broad-spectrum killing” to “precision reprogramming”

This paper redefines the trajectory of GC development by positing that the evolution from *H. pylori* infection to metastatic GC is not merely a result of accumulated gene mutations, but rather the evolutionary activation of a “survival switch.” Although *H. pylori* CagA serves as the initial “igniter,” disrupting epithelial integrity, the disease’s true lethality is derived from the subsequent “transfer” of control to epigenetic mechanisms (such as METTL3-mediated methylation) and the inflammatory microenvironment. This “Hit-and-Run” mechanism enables tumor cells to persist in a drug-resistant, autonomous “mixed E/M” state even after bacterial eradication through epigenetic scarring.

This mechanistic insight reveals a critical flaw in the current “eradication + chemotherapy” clinical paradigm: the concentration of existing cytotoxic drugs is primarily on proliferating cells, which may result in the selection of more plastic, drug-resistant subclones and the failure to eliminate dormant stem cells. Future research and treatment strategies must accomplish breakthroughs in the following three dimensions in order to overcome this clinical impasse:

Initially, there must be an analysis of heterogeneity and a reshaping of disease models. In order to accurately replicate the spatiotemporal process of *H. pylori*-induced EMT within a physiological context that preserves the immune microenvironment, it is imperative that we forsake traditional two-dimensional cell lines and transition to the use of patient-derived organoids (PDOs) and organ-on-a-chip technology. Additionally, in order to accurately represent the intricate inflammatory ecosystem delineated in this review, particularly the interplay between TAMs, TANs, and CAFs, research must increasingly depend on humanized patient-derived xenografts (hPDXs) and genetically engineered mouse models (GEMMs). These advanced in vivo models are essential for determining whether ‘epigenetic scars’ can legitimately maintain the mesenchymal state within a fully immunocompetent stroma following the clearance of *H. pylori*. Simultaneously, by integrating spatial transcriptomics and single-cell sequencing (scRNA-seq), we can identify the “zero point” of CagA translocation and the emergence of highly plastic subsets at single-cell resolution. This allows us to identify early biomarkers that are capable of inhibiting this process.

Subsequently, a paradigm shift in clinical treatment strategies is necessary, transitioning from a singular “search and destroy” approach to a biomarker-based “wake-up and kill” sequential treatment strategy. This requires that clinical trial designs evolve beyond simple inhibition and adopt a “push-and-strike” approach. During the induction phase, epigenetic regulators or EMT reversal agents are employed to “unlock” the stromal resistance state of cells, thereby causing them to regain their epithelial phenotype and chemotherapy sensitivity. Subsequently, a high-intensity cytotoxic attack is administered during the transient “treatment window,” specifically targeting the metabolically unstable transition state, in order to eradicate cells before they regain the capacity for colonization.

Nevertheless, a comprehensive assessment of the clinical feasibility of this sequential approach is necessary. First and foremost, the precise spatiotemporal ‘vulnerability window’ in humans is a significant pharmacokinetic challenge, as mistimed MET induction poses a risk of catastrophic metastatic colonization. Secondly, the dependence on continuous, real-time liquid biopsies for patient stratification necessitates a high level of standardization and poses substantial cost barriers to widespread clinical adoption. Finally, although nanocarrier-based RNA delivery platforms exhibit significant preclinical potential for targeting ‘undruggable’ EMT-TFs, the regulatory approval pathways regarding their systemic toxicity, off-target effects, and biodistribution in advanced GC patients continue to be a significant challenge. Multidisciplinary clinical trials that are explicitly designed around adaptive, biomarker-driven endpoints will be necessary to surmount these translational barriers.

In conclusion, therapeutic interventions that target the “survival switch” in GC are essentially a game of converting pathogen-driven survival advantage into clinical vulnerability. Our objective is to achieve more than mere tumor suppression by combining precise molecular-level reprogramming with sequential clinical-level attacks. We are committed to profoundly dismantling the defense mechanisms that drive GC recurrence and metastasis.
